# Mechanisms of tumor-associated macrophages affecting the progression of hepatocellular carcinoma

**DOI:** 10.3389/fphar.2023.1217400

**Published:** 2023-08-17

**Authors:** Yi Yuan, Dailin Wu, Jing Li, Dan Huang, Yan Zhao, Tianqi Gao, Zhenjie Zhuang, Ying Cui, Da-Yong Zheng, Ying Tang

**Affiliations:** ^1^ Guangzhou University of Chinese Medicine, Guangzhou, Guangdong, China; ^2^ Department of Psychiatry, The Third Affiliated Hospital of Guangzhou Medical University, Guangzhou, Guangdong, China; ^3^ Department of Oncology, The First Affiliated Hospital of Guangzhou University of Chinese Medicine, Guangzhou University of Chinese Medicine, Guangzhou, Guangdong, China; ^4^ Department of Hepatology, TCM-Integrated Hospital of Southern Medical University, Guangzhou, Guangdong, China; ^5^ Department of Hepatopancreatobiliary, Cancer Center, Southern Medical University, Guangzhou, Guangdong, China; ^6^ Science and Technology Innovation Center, Guangzhou University of Chinese Medicine, Guangzhou, Guangdong, China

**Keywords:** hepatocellular carcinoma, tumor-associated macrophages, tumor microenvironment, cancer stem cells, carcinoma cell proliferation, invasion and migration, angiogenesis, hepatic fibrosis

## Abstract

Tumor-associated macrophages (TAMs) are essential components of the immune cell stroma of hepatocellular carcinoma. TAMs originate from monocytic myeloid-derived suppressor cells, peripheral blood monocytes, and kupffer cells. The recruitment of monocytes to the HCC tumor microenvironment is facilitated by various factors, leading to their differentiation into TAMs with unique phenotypes. TAMs can directly activate or inhibit the nuclear factor-κB, interleukin-6/signal transducer and signal transducer and activator of transcription 3, Wnt/β-catenin, transforming growth factor-β1/bone morphogenetic protein, and extracellular signal-regulated kinase 1/2 signaling pathways in tumor cells and interact with other immune cells via producing cytokines and extracellular vesicles, thus affecting carcinoma cell proliferation, invasive and migratory, angiogenesis, liver fibrosis progression, and other processes to participate in different stages of tumor progression. In recent years, TAMs have received much attention as a prospective treatment target for HCC. This review describes the origin and characteristics of TAMs and their mechanism of action in the occurrence and development of HCC to offer a theoretical foundation for further clinical research of TAMs.

## Introduction

Hepatocellular carcinoma (HCC) accounts for roughly 90% of all cases of primary liver carcinoma, making it the most prevalent form. It ranked as the 6th most common carcinoma and the 3rd most common cause of cancer-related fatalities in 2020. It was projected that HCC caused 906,000 new cases and 830,000 deaths worldwide due to HCC (Sung et al., 2021; Ding et al., 2022). HCC is associated with inflammation and can be caused by various factors, including environmental and genetic risk factors (Giraud et al., 2021). Chronic infection caused by hepatitis C virus (HCV), hepatitis B virus (HBV), and non-alcoholic fatty liver disease/non-alcoholic steatohepatitis (NAFLD/NASH) are the leading etiologies of liver cirrhosis, which can significantly increase the likelihood of progression to HCC (Kew, 2014). Chronic liver injury triggers reparative mechanisms aimed at restoring its form and function. However, this persistent inflammation can lead to ongoing regenerative repair of hepatocytes, which may contribute to the emergence and advancement of HCC (Endig et al., 2016; Gao et al., 2019). The malignant level of a tumor is related to the characteristics of cancerous cells and different components in the tumor microenvironment (TME) (Wang et al., 2014; Ge and Ding, 2020). These constituents have a pivotal function in connecting inflammatory mediators and tumors (Chen et al., 2022). The TME is a significant site for the interaction between carcinoma cells and the human immune system. It contains neoplastic cells, immune cells, blood vessel cells, tumor-associated fibroblasts, other non-tumor cells, extracellular matrix, and various cytokines (Li et al., 2018). In recent years, the immune microenvironment has gained an increasing focus, and it is indicated that the TME can facilitate tumor progression. Research has indicated that the high infiltration of tumor-associated macrophages (TAMs) in liver cancer is strongly tied to a negative prognosis for patients (Li et al., 2018b). Compared to other organs, the liver has a greater percentage of macrophages (van der Heide et al., 2019). Kupffer cells (KCs), as a stationary tissue-resident macrophage subset of the liver, are positioned in the blood sinuses, which is pivotal in maintaining homeostasis (Lopez et al., 2011; Scott et al., 2016). Inflammation in the liver or depletion of KCs leads to recruiting monocyte-derived macrophages into the stem tissue (Mossanen et al., 2016; Surewaard and Kubes, 2017). Tumor cells secrete different cytokines that can induce monocyte differentiation into TAMs, thereby altering the functional phenotype of macrophages (Mφ) in the TME (Rabold et al., 2017). These mutual transformations are a common occurrence in cancer and are correlated with tumor occurrence and development. The dysregulation of the two types of TAMs polarization is frequently involved in the development of pathological complications (Qian and Pollard, 2010). Interactions between tumor cells, TAMs, and other immune cells in the immune microenvironment affect the proliferation, invasion, migration, liver fibrosis, and immune killing of tumor cells through the secretion of cytokines and exosomes and changes in the expression of related proteins, ultimately affecting the progression of HCC. The mechanisms and signaling pathways involved are very complex. Therefore, this article provides a review of the definition, source, and polarization of TAMs and the mechanisms of interaction among TAMs, tumors, and other immune cells in HCC.

### Definition of TAMs in HCC

Mφ are one of the most significant components of the HCC TME. The liver, as an immune-exempt organ, contains a significant number of Mφ, including resident KCs and recruited Mφ (MacParland et al., 2018; Mattos Â et al., 2022). Based on protein expression, secreted cytokines, and function, TAMs are typically divided into two subgroups (Zhou et al., 2022): classical activated TAMs (M1-TAMs) and alternative activated TAMs (M2-TAMs) (Lin et al., 2019). M1-TAMs are mainly present in the tumor adjacent tissue, while M2-TAMs are mainly present in the liver cancer tissue (Luo et al., 2017; Englinger et al., 2019). M1-TAMs promote more inducible nitric oxide synthase (iNOS) and mainly secrete interleukin (IL)-1β, tumor necrosis factor (TNF)-α, highly expressed cluster of differentiation (CD)80, and CD86. M2-TAMs decreased the protein expression of CD80 and CD86, while elevating the expression of CD163, CD206, and arginase (Arg)-1. They also secreted less TNF-α and IL-1β, but more transforming growth factor (TGF)-β and IL-10 (Dong et al., 2016; Minami et al., 2018; Li et al., 2021; Sen et al., 2022). Moreover, CD68 was identified as a reliable marker for pan-Mφ or M1-TAMs (Minami et al., 2018). Additionally, the M2-TAMs could be further classified into four subsets (M2a, M2b, M2c, and M2d) based on the type of stimulus (Yang et al., 2019). When stimulated with interferon-gamma (IFN-γ) or IFN-γ integrated with lipopolysaccharide (LPS), Mφ become classically activated Mφ or M1-TAMs. Unlike M1-TAMs, which are polarized by proinflammatory stimuli, IL-4, IL-13, IL-10, and TGF-β can induce Mφ polarization into M2-TAMs, also known as alternatively activated Mφ. These Mφ highly express mannose receptor (MR) and Arg-1, which are connected to anti-inflammatory Th2 immunoreaction and can promote HCC progression (Yang et al., 2018a). Both M1-TAMs and M2-TAMs can be converted into each other in certain conditions and with specific stimuli (Xiang et al., 2021). M1-TAMs appear to eliminate HCC cells in the primary stages of tumorigenesis, but as tumor progression advances, M1-TAMs are replaced by M2-TAMs (Yao et al., 2018). Specifically, Mφ that infiltrate tumors tend to exhibit M2 phenotypes that promote tumor growth, rather than M1 phenotypes that have antitumor effects (Chen et al., 2018). Currently, many studies have found that the phenotype and function of TAMs are very complex, and it is limited to classify TAMs simply as M1 and M2 phenotypes. Therefore, there are many studies to distinguish different TAM subgroups through the special phenotype expression of TAMs and to investigate the impact of different subgroups on the progression of HCC. Intra-tumoral TAMs in HCC are often characterized by low expression of CD169 and high expression of CD204. Reversing this trend can improve patient prognosis (Li et al., 2017). The pro-inflammatory response induced by a high-fat diet leads to an increase in TNF-α^+^ Mφ infiltration, promoting the early onset of HCC (de Oliveira et al., 2019). Chemokine (CC motif) receptor (CCR)2^+^ monocytes and triggering receptor expressed on myeloid cells (TREM) 2^+^ Mφ in the TME can terminally differentiate to matrix metalloproteinase (MMP)9^+^ TAMs to promote the progression of HCC by stimulating the peroxisome proliferator-activated receptor (PPAR)γ signal (Lu et al., 2022). Siglec-10^hi^ TAMs have characteristics and functions similar to M2-TAMs and can inhibit the function of CD8^+^ T cells (Xiao et al., 2021). These phenotypic TAMs play an immunosuppressive role in the TME of HCC. By contrast, CD38^hi^ Mφ in the TME can secrete more IFN-γ, which helps CD8^+^ T cells to kill tumors (Ng et al., 2020). In addition, forkhead box O1 (FOXO1) expressed in TAMs inhibits the expression of IL-6 through the interferon regulatory factor (IRF)-1/nitric oxide (NO) axis and ultimately suppresses the progression of HCC (Cui et al., 2023). Furthermore, loss of FOXO1 leads to a decrease in major histocompatibility complex (MHC)-II expression, which weakens the antigen-presenting ability of TAMs and hinders the ability of immune cells to kill tumor cells. The hypoxic microenvironment in HCC induces the downregulation of FOXO1 expression in TAMs (Yang et al., 2018b). Typically, the majority of TAMs within the TME display an immunosuppressive type, and these TAMs promote immune suppression and facilitate tumor progression (Allavena et al., 2008). To further elaborate, TAMs refer to immunosuppressive Mφ in the HCC TME in a narrow sense.

### Origin of TAMs in HCC

TAMs originate from peripheral blood monocytes, M-MDSCs, and KCs. Under normal conditions, Mφ can be distinguished from peripheral monocytes in tissues. However, during inflammation and cancer, peripheral monocytes derived from the bone marrow (BM) were one of the principal sources of Mφ, particularly TAMs. Under the stimulation of inflammatory and tumor signals, circulating monocytes can mobilize and infiltrate in the TME, where they transform into tissue Mφ (Shi and Pamer, 2011; Nowarski et al., 2013). HCC produces extracellular vesicles (EVs) containing PKM2, which induce phosphorylation of the signal transducer and activator of transcription 3 (STAT3) in monocytes, promoting their differentiation into TAMs (Hou et al., 2020). Chronic liver injury is closely related to the infiltration of M2-TAMs derived from monocytes (Delire et al., 2018). Most TAMs are derived from CCR2 ^+^ monocytes in BM (Cheng et al., 2022). Monocytes are attracted to the TME through the (CC motif) ligand 2 (CCL2)/CCR2 axis, transformed into TAMs, polarized toward M2-TAMs, and involved in the HCC progression (Sahin et al., 2010; Li et al., 2017). CCR2^+^ myeloid cells are necessary for senescence surveillance, and CCR2 ablation leads to HCC outgrowth (Eggert et al., 2016). The TME causes the overexpression of dynamin-related protein 1 (Drp1) in the HCC cells, which stimulates mitochondrial fission and induces cytoplasmic mitochondrial DNA (mtDNA) stress, activating the TLR9-mediated NF-κB signaling pathway and promoting the nuclear translocation of phosphorylated P65, thereby promoting the secretion of chemokine CCL2 (Bao et al., 2019). Additionally, the expression level of APOBEC3B (A3B) is upregulated in HCC cells, and A3B interacts with polycomb repressive complex 2 (PRC2), leading to reduced occupancy of H3K27me3 on the CCL2 promoter to recruit massive TAMs (Wang et al., 2019). Meanwhile, the expression of CCL2 by tumor cells is also regulated by Yes-associated protein (YAP)/TEA domain family member 4 (TEAD4) dependence (Thomann et al., 2021). Blocking CCL2/CCR2 and cysteine-X-cysteine motif chemokine ligand (CXCL)1/CXCR2 can inhibit the infiltration levels of Mφ and neutrophils in the TME (Tian et al., 2022). Meanwhile, the high expression of hsa_circ_0003410 in HCC induces the secretion of CCL5, activating and recruiting M2-TAMs (Cao et al., 2022). The upregulation of MMP-21 expression in tumor cells promotes the secretion of CCL14, which induces the recruitment of monocytes through binding to CCR1 on these cells (Zhou et al., 2022). CSF1 is a chemotactic factor for monocytes and can facilitate recruitment of monocytes and polarization of Mφ to M2-TAMs (Zhu et al., 2019; Jiang et al., 2022). The colony-stimulating factor 1 receptor (CSF-1R) is mainly expressed by TAMs and monocyte-restricted cells (Jiang et al., 2020). TAMs promote Mφ migration by upregulating the expression of M-CSFR and CXCR4 through hypoxia inducible factor (HIF)-2α mediation (Imtiyaz et al., 2010). Prostaglandin E (PGE)_2_ secreted by TAMs stimulates the expression of UHRF1 in tumor cells, which in turn induces the upregulation of CSF1 expression (Zhang et al., 2022). IL-1β secreted by TAMs induces the upregulation of solute carrier family 7 member 11 (SLC7A11) expression in HCC. SLC7A11 upregulation promotes HIF-1α expression by reducing α-ketoglutarate (αKG) levels, which in turn promotes the expression of programmed death ligand 1 (PD-L1) and CSF1 in tumor cells (He et al., 2021). Circular RNA (circRNA) CircASAP1 competes with tumor suppressor miRNAs (miR-326 and miR-532-5p) that target CSF1, also promoting the expression of CSF1 (Hu et al., 2020). The overexpression of stanniocalcin-1 (STC1) in tumor cells inhibits the expression of monocyte chemokine receptors chemokine CCR2, CCR4, and colony-stimulating factor 1 receptor (CSF1R), thereby suppressing the recruitment of monocytes to the TME (Leung and Wong, 2020).

TAMs can also originate from myeloid-derived suppressor cells (MDSCs), which also come from BM. MDSCs are composed of two major groups: granulocytic or polymorphonuclear (PMN-MDSCs and Ly6C^−^ Ly6G^+^) and monocytic (M-MDSCs and Ly6C^+^ Ly6C^−^) (Tcyganov et al., 2018). PMN-MDSCs share similar characteristics with neutrophils, while M-MDSCs are more akin to monocytes (Gabrilovich et al., 2012). In the context of tumor tissues, M-MDSCs have the ability to quickly differentiate into TAMs and inflammatory dendritic cells (DCs).

Although bone marrow-derived monocytes were previously regarded as the only origin of TAMs, recent research studies indicated that tissue-determined Mφ can infiltrate tumors and transform into TAMs in specific tumors (Zhu et al., 2017; Conte, 2022). Liver-resident Mφ, like KCs (Matsuda and Seki, 2020), can stem from erythro-myeloid progenitors (EMPs) in the embryonic liver or the yolk sac, which express the Mφ CSF1R (Laviron and Boissonnas, 2019; Cheng et al., 2022). These Mφ can self-renew, but recent studies have shown their input from the bone marrow as well (Gomez Perdiguero et al., 2015; Hoeffel et al., 2015; Scott et al., 2016). As one of the subsets of TAMs, KCs can also facilitate the progression of HCC while participating in anti-tumor immunity (Yang et al., 2011). Signals from the local microenvironment stimulate immunogenic KCs and induce their functional differentiation. Danger signals in HCC can activate the inflammatory regulation of KCs and promote drawing immune cells to the liver. The production of CCL2 by tumor cells leads to the absence of embryonic KCs in tissue and promotes the infiltration of monocyte-derived KCs and immature monocytes (M0) (Thomann et al., 2021; Vanderborght et al., 2023). Myeloid precursor cells are recruited to infiltrate HCC, and the TME blocks its maturation process. The loss of Shp2 expression in KCs results in apoptosis of KCs and exacerbate the recruitment and differentiation of CCR2^+^ monocytes into TAMs, reshaping the immunosuppressive microenvironment (Du et al., 2023). However, some studies suggest that activated KCs also produce TNF-a, and IL-1, inducing the expression of IL-8 in HCC cells and promoting Mφ recruitment (Thornton et al., 1991). Expressing neurotensin (NTS) in tumor cells activates the mitogen-activated protein kinase (MAPK)/nuclear factor (NF)-κB signaling pathway, inducing the production of IL-8 (Xiao et al., 2018). In addition, the upregulation of AlkB homolog 5 (ALKBH5) promotes the upregulation of MAP3K8 expression in tumor cells, which induces the phosphorylation of the c-Jun N-terminal kinase (JNK)/extracellular signal-regulated kinase (ERK) pathway, resulting in the upregulation of IL-8 expression. IL-8 promotes the recruitment of PD-L1^+^TAMs and polarization of M2-TAMs (You et al., 2022). TAMs originate not only from the recruitment of monocytes or MDSCs but also from the proliferation of TAMs themselves residing in the TME. Tumor cells stimulates TAMs to secrete granulocyte-macrophage colony stimulating factor (GM-CSF), which induces the upregulation of A2A receptor expression in TAMs. This process synergistically stimulates the proliferation of TAMs in coordination with small molecules of adenosine produced by tumor cells, leading to increased infiltration of TAMs (Wang et al., 2021).

**FIGURE 1:**
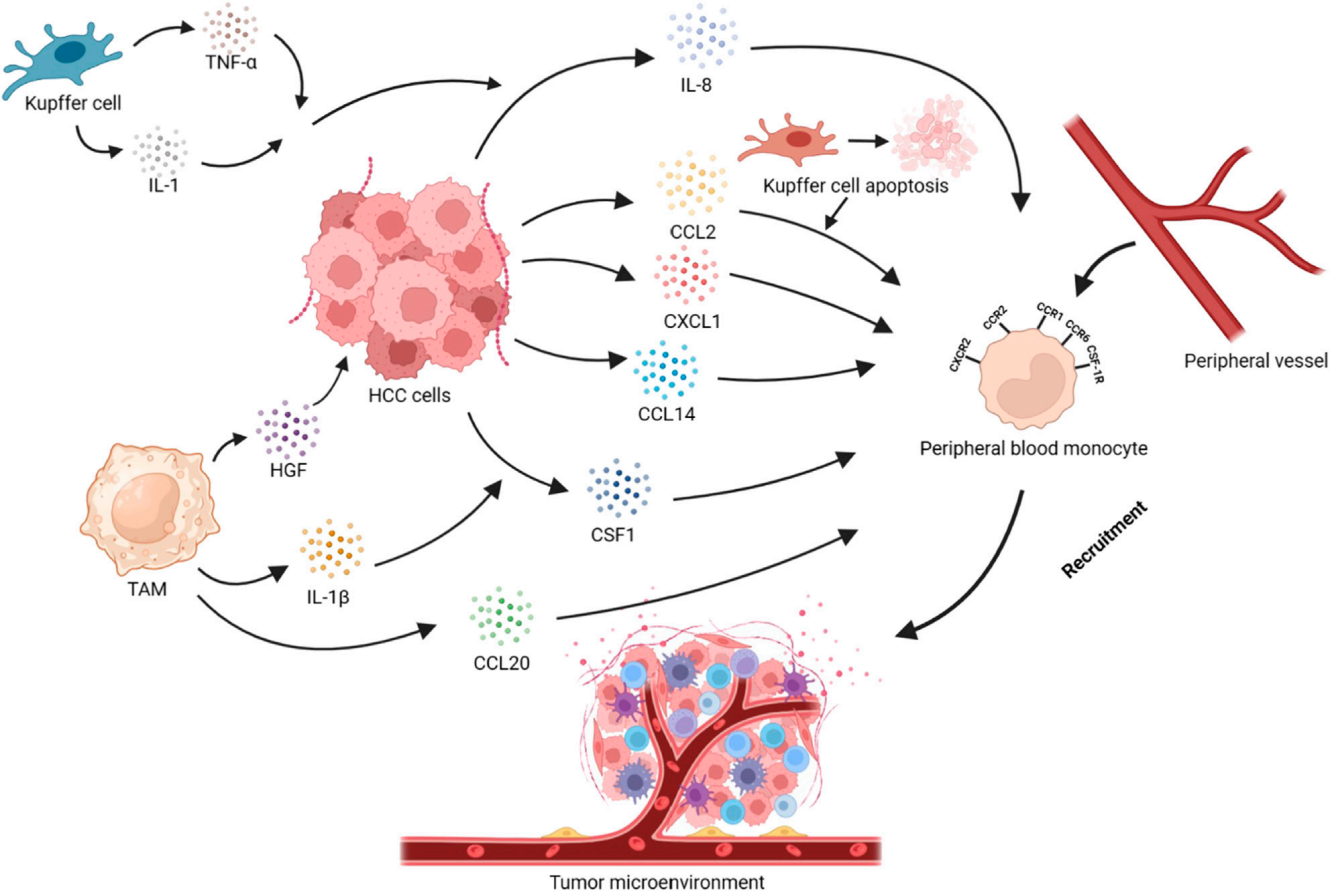
Recruitment of peripheral blood monocytes in HCC. Tumor cells secrete cytokines or chemokines such as CCL2, CXCL1, CCL14, CCL20, and CSF1, which recruit monocytes into the TME via the CCL2/CCR2, CXCL1/CXCR2, CCL14/CCR1, and CSF1/CSF-1R axes. The apoptosis of KCs promotes the recruitment of monocytes through the CCL2/CCR2 axis. IL-1, and TNF-α secreted by KCs act on HCC cells, promoting the secretion of IL-8 and, thus, recruiting monocytes. IL-1β secreted by TAMs can promote HCC cells to secrete CSF1. HGF secreted by TAMs is closely related to monocyte recruitment in HCC cells. At the same time, TAMs can also secrete CCL20 to achieve self-recruitment.

Cancer-associated fibroblasts (CAFs) express endosialin (CD248/TEM1) and interact with CD68, inducing the recruitment of Mφ. Meanwhile, this process induces GAS6 expression in CAFs, promoting the polarization of TAMs toward M2-TAMs (Yang et al., 2020). M2-TAMs secrete the hepatocyte growth factor (HGF) to help recruiting Mφ, and promote the infiltration of M2-TAMs (Dong et al., 2019). Infiltrating monocytes in HCC stimulate the production of TNF-α through the NF-κB signaling pathway, inducing the expression of their own c-Met. When interacting with HGF, this enhances their migration ability (Zhao et al., 2015). Autophagy inhibition in Mφ induces self-recruitment through the activation of the CCL20/CCR6 signaling pathway (Gao et al., 2023). However, in HCC cells, interferon regulatory factor (IRF8) inhibits the transcription of activator protein-1(AP1) signal (c-fos), resulting in a decrease in the expression of CCL20 and the inhibition of TAMs recruitment, thereby suppressing tumor progression (Wu et al., 2022). In M1-TAMs, the extracellular matrix protein SPON2 activates RhoA/Rac1-Hippo signaling through α4β1 integrin, leading to F-actin reorganization. The upregulation of F-actin expression inhibits large tumor suppressor kinase 1 (LATS1) phosphorylation and promotes YAP nuclear translocation, promoting the recruitment of M1-TAMs and inhibiting HCC progression. The specific mechanism by which YAP affects Mφ migration remains unclear (Zhang et al., 2018). However, the high expression of YAP in tumor cells induces an increase in IL-6 secretion, which plays an opposite role in tumor progression (Zhou et al., 2018). Hepatic stellate cells activated within tumors exhibit high expression of fatty acid-binding protein 4 (FABP4), inducing NF-κB nuclear translocation and promoting the secretion of IL-6 and IL-1A (Chiyonobu et al., 2018). By activating the IL-6-IL6R/STAT3 signaling pathway in Mφ, IL-6 induces the recruitment of Mφ and polarization toward the M2 phenotype (Guo et al., 2017; Yang and Xing, 2021). Hypoxia leads to the expression of high-mobility group box 1 (HMGB1), which induces the infiltration of TAMs and an increase in IL-6 expression through HIF-1α (Jiang et al., 2018). (Figure 1)

In the TME, tumor cells, KCs, TAMs with different phenotypes, hepatic stellate cells, and CAFs can produce multiple cytokines that promote the generation of TAMs. These chemokines play a significant role in recruiting and infiltrating macrophages into tumor tissues. The recruitment and chemotaxis of TAMs play a promoting part not only in liver cancer (Shieh et al., 2009) but also in many malignant tumors. How to inhibit the recruitment and chemotaxis of Mφ has gradually become one of the hotspots in the therapy of HCC.

### TME affects the polarization of TAMs

**FIGURE 2:**
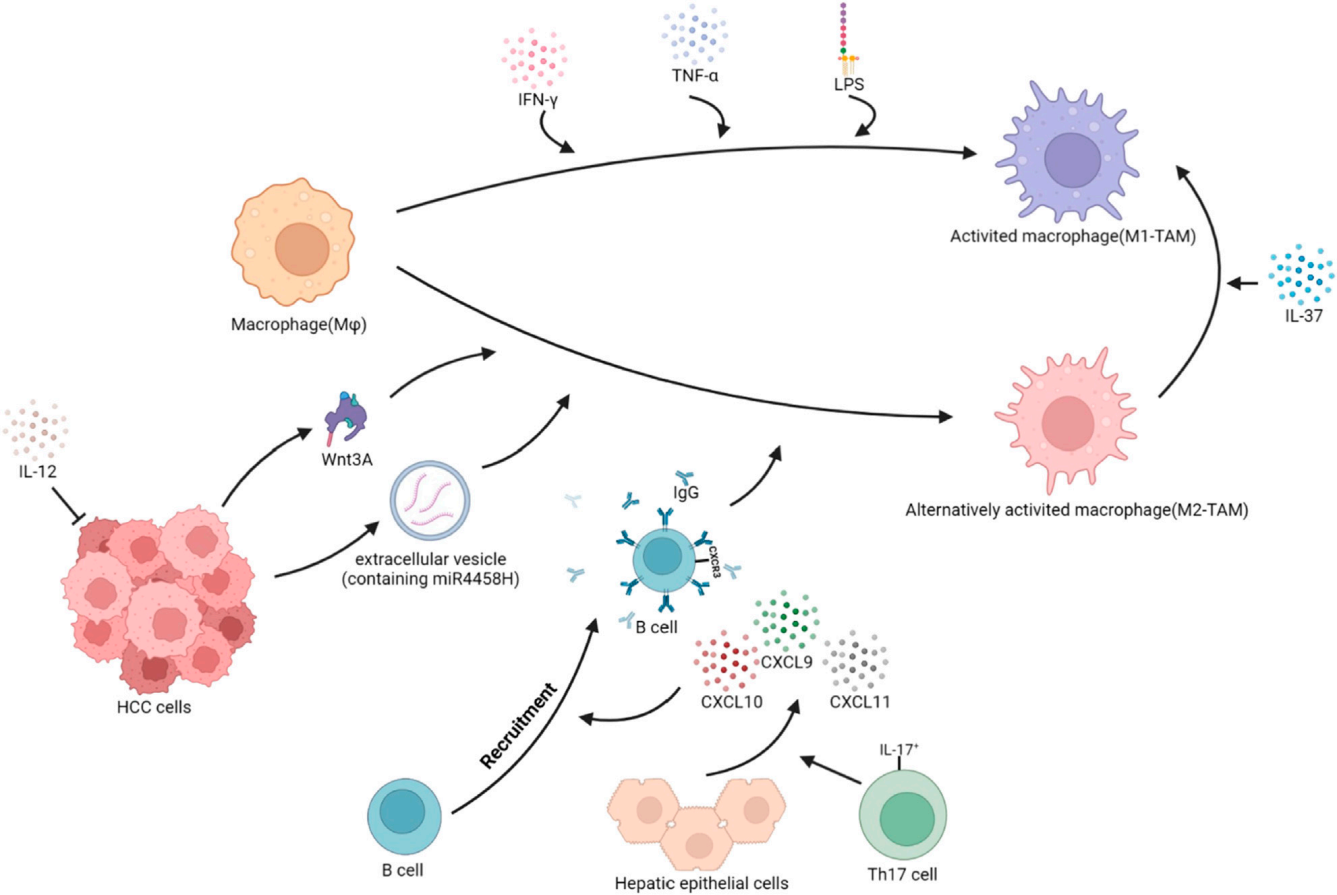
Polarization of TAMs in HCC. The local cytokine environment can determine the polarization of macrophages. Based on protein expression, secreted cytokines, and function, TAMs are typically divided into two subgroups: classical activated TAMs (M1-TAMs) and alternative activated TAMs (M2-TAMs). HCC cells secrete Wnt3A ligands and extracellular vesicles containing miR4458H, which can induce the polarization of Mφ toward M2-TAMs. IL-17^+^ cells stimulate hepatocytes to secrete CXCL9, CXCL10, and CXCL11, promoting the recruitment of CXCR3^+^B cells and inducing the polarization of Mφ towards M2-TAMs through the IgG pathway. IL-12, which has anti-tumor effects, reduces secretion and indirectly promotes the polarization of Mφ toward M2-TAMs. Conversely, when stimulated with IFN-γ, or IFN-γ integrated with LPS, Mφ become classically activated Mφ, or M1-TAMs. Additionally, in the TME, IL-37 can induce M2-TAMs to reverse into M1-TAMs.

The tumor immune microenvironment contains cytokines and extracellular vesicles secreted by tumor cells, which can induce the polarization of TAMs. The polarization of TAMs is also related to the transport of zinc and ferritin, as well as the metabolism of carbohydrates and lipids. As liver cancer advances, pro-tumorigenic factors stimulate tissue-resident Mφ, leading to a phenotypic shift and the development of TAMs (Cheng et al., 2022). In HCC, IL-37 is highly expressed in M1-TAMs and lowly expressed in M2-TAMs. IL-37 promotes the polarization of M2-TAMs toward M1-TAMs in HCC by inhibiting IL-6/STAT3 signaling in TAMs (Zhang et al., 2020a). The high expression of B7 homolog 3 (B7-H3) in HCC polarizes TAMs toward the M2 direction by the activation of STAT6 signaling (Kang et al., 2015). The Wnt ligand produced by tumor cells activates the Wnt/β-catenin signaling pathway in Mφ, promoting the nuclear translocation of β-catenin and transcriptional upregulation of C-MYC, inducing M2-TAMs polarization (Yang et al., 2018). In HCC cells, the lncRNA LINC00662 can competitively bind to miR-15a, miR-16, and miR-107, upregulating WNT3A secretion (Tian et al., 2020). Tumor cells secrete EVs containing miR4458H, which induces the upregulation of Arg1 expression in TAMs, promoting M2-TAMs polarization (Ye et al., 2023). Receptor-interacting protein 140 (RIP140) is overexpressed in TAMs, which inhibits the alternative activation of Mφ and has an inhibitory effect on the progression of HCC by suppressing the NF-κB/IL-6 axis (Hu et al., 2017). IL-17^+^ cells induce epithelial cells to secrete CXCL9, CXCL10, and CXCL11, recruiting CXCR3^+^B cells and promoting their maturation. CXCR3^+^B cells polarize M2b through IgG signaling (Liu et al., 2015). TREM1 is highly expressed in M2 TAMs. The downregulation of TREM1 expression reverses M2-TAMs into M1-TAMs by inhibiting the PI3K/AKT/mTOR signaling pathway (Chen et al., 2021). HCC produces HMGB1, which activates the Toll-like receptor 2 (TLR2)/NADPH oxidase 2 (NOX2)/autophagic axis in TAMs. NOX2 induces reactive oxygen species (ROS) production, which induces autophagy. The autophagic process degrades NF-κB p65 through the p62/SQSYM1 signaling pathway, inhibiting the secretion of IL-12 (which has anti-tumor effects) and promoting polarization of Mφ toward the M2 phenotype. Meanwhile, TLR2 mediates the phosphorylation and activation of ERK1/2, promoting the degradation process of NF-κB p65 and synergistically promoting the polarization of TAMs toward the M2 direction (Chang et al., 2013; Shiau et al., 2020). The expression loss of liver cell zinc finger protein Miz1 activates the NF-κB signaling pathway, promoting Mφ polarization toward the pro-inflammatory phenotype and promoting the progression of HCC (Zhang et al., 2021). Zinc/iron-regulated transporter-like protein (Zip) 9, which is associated with zinc uptake, is highly expressed in M2-TAMs of HCC and promotes M2 polarization by activating the STAT6 signaling pathway, while inhibiting M1 polarization by inhibiting IκBα/β signaling (Gou et al., 2022). The ferrous iron content in M1-TAMs is higher than in M2-TAMs. The high expression of transferrin in HCC cells leads to insufficient iron uptake by TAMs, which promotes the polarization of M2-TAMs through the upregulation of HIF-1α (Sun et al., 2021). (Figure 2)

Abnormalities in glucose and lipid metabolism in HCC affect the function and polarization of TAMs, accelerating tumor progression. In HCC, aerobic glycolysis promotes the activation of M1-TAMs, but the hypoxic microenvironment and insufficient glucose in the tumor lead to an increase in fatty acid oxidation (FAO) and a decrease in aerobic glycolysis. This promotes the expression of ROS in TAMs and induces TAMs to transform into an M2 phenotype through nuclear factor (erythroid-derived 2)-like 2 (Nrf2) mediation (Feng et al., 2018). The hypoxic microenvironment of HCC leads to abnormal glucose metabolism in tumor cells, with an increase in anaerobic glycolysis and lactate secretion. However, even in the presence of sufficient oxygen, tumor cells preferentially undergo anaerobic glycolysis, exhibiting the Warburg effect. The overexpression of pH-regulating molecule V-ATPase in M2-TAMs induces hypoxic and acidic metabolism, reshaping the immunosuppressive microenvironment (Kuchuk et al., 2018). The expression of receptor-interacting protein kinase 3 (RIPK3) in HCC is closely related to glucose and lipid metabolism. The downregulation of RIPK3 activates the PPAR signaling pathway to promote fatty acid metabolism and FAO, inducing M2 polarization, which depends on the mediation of ROS-caspase1 (Wu et al., 2020). The downregulation of sirtuin (SIRT)5 expression in liver cancer induces the production of bile acids (BAs) (Sun et al., 2022). Abnormal BAs metabolism induces M2-TAMs polarization, and the excessive production of BAs reshapes the immunosuppressive microenvironment. On the contrary, (SIRT)1 promotes the polarization of Mφ toward the M1 direction, inhibiting the progression of HCC (Zhou et al., 2019). Therefore, targeting these processes and signaling pathways can alter the polarization process of TAMs in the TME and reverse the immune-suppressive microenvironment.

### TAMs impact the malignant biological behaviors of HCC

Many studies have indicated that Mφ infiltrating HCC affect the progression of the disease by secreting cytokines, chemokines, and matrix metalloproteases and releasing EVs containing non-coding RNA that have an impact on HCC cells, including proliferation, apoptosis, angiogenesis, cancer stem cell properties, invasion and migration abilities, liver fibrosis progression, and the immune microenvironment. Proteins, lipids, and RNAs are contained in exosomes, which mediate intercellular communication between different cell types, thus affecting the progression of cancer (Colombo et al., 2014). There have been reports that demonstrated that some signaling pathway-mediated crosstalk between Mφ and HCC cells cause M2-TAM polarization, leading to HCC growth, migration and metastasis, such as IL-6/STAT3, Wnt/β-catenin, and NF-κB (Mano et al., 2013; Yan et al., 2015; Yang et al., 2018).

### TAMs could affect the level of cancer stem cells in HCC

The transformation of HCC cells into stem-cell like entities is facilitated by alterations in the TME. Consequently, HCC cells can acquire characteristics including self-renewal, differentiative capacity, and rejection of treatment, which are the stemness of cancerous cells (DeLuca and Saleh, 2023). The multiplication of HCC cells is corresponding to the cancer stem cells (CSCs). Patients with high stemness scores presented a higher infiltration level of TAMs, implying the presence of an immunosuppressive microenvironment (Zhang et al., 2022). Furthermore, CSCs high-risk patients with CD133 as a marker may exhibit immune liver homeostasis disorder (Yu et al., 2020). In addition, there is a positive interrelation between the infiltration of CD68^+^ TAMs and the number of OV6^+^ CSCs or EpCAM^+^ CSCs in HCC (Yao et al., 2016). The infiltration of these three types of cells is related to inferior overall survival (OS) and progression-free survival (PFS) of individuals with HCC (Fan et al., 2014; Wang et al., 2022). M2-TAMs could secrete CXCL1 and CXCL2 as potential paracrine factors. In addition to their CSCs properties, CXCL1 and CXCL2 were found to stimulate the transcription of BCL-2 while suppressing the transcription of BAD and BAX. BCL-2 is an optimistic target for eliminating CSCs in HCC (Wang et al., 2022). The targeting of CD90 by miR-125a/b was found to have a central impact on HCC CSCs. TAMs exosomes with reduced ranks of miR-125a and miR-125b facilitate HCC cell multiplication and stemness (Wang et al., 2019). It was observed that CD163^+^ TAMs expressed arachidonic acid 5-lipoxygenase (5-LOX) and generated LTB4 and LTC/D/E4 in a mouse model of HCC. These molecules were found to facilitate oncogenesis and CSCs properties by activating the phosphorylation of ERK1/2 and CSCs-associated genes (Nosaka et al., 2023). *In vitro* research studies have confirmed that CD44^+^ cells derived from both HCC specimens and cell lines exhibit CSCs actions. Furthermore, the secretion of IL-6 by TAMs were found to facilitate the expansion of CSCs in HCC and boost the migration process (Mano et al., 2013; Wan et al., 2014). It is indicated that IL-6 produced by cocultured Mφ activated STAT3 signaling of Hep1-6 cells induced the invasion of HCC cells in the hypoxic environment (Jiang et al., 2018). TAMs can secrete S100 calcium-binding protein A9 (S100A9) to induce the pro-inflammatory milieu in HCC. This upregulation occurs in a Ca^2+^-dependent manner and activates the NF-κB pathway via the advanced glycosylation end product-specific receptor (AGER). This activation enhances the stemness of HepG2 and MHCC-97H cells (Wei et al., 2021). Therefore, TAMs promote their M2 polarization and proliferation by expressing relevant proteins through paracrine cytokines and EVs.

CSCs existing in HCC are closely bound to enhanced invasion and migration, which contributes to the aggressive nature of this cancer, besides unrestrained cell replication in cancer. The releasement of TNF-α by M2-TAMs has been shown to facilitate EMT and CSCs via the Wnt/β-catenin signaling (Chen et al., 2019). Additionally, M2-TAMs secrete CCL17 to enhance the stemness and epithelial–mesenchymal transition (EMT), as well as TGF-β1 and Wnt/β-catenin signaling transduction of HCC cells (Zhu et al., 2016). In addition to cytokines, EVs from M2-TAMs could also affect the CSCs-like characteristics and invasive migration ability of HCC cells (Liang et al., 2016; Cheng et al., 2022). The miR-17-92 cluster, deriving from EVs of M2-TAMs, was observed to disrupt balance in the TGF-β1/bone morphogenetic protein (BMP)-7 signaling in cancerous cells. This was achieved by inhibiting the post-translational ubiquitylation of activin A receptor type 1 (ACVR1) and inducing the post-transcriptional silencing of TGF-β type II receptor (TGFBR2) through the targeting of Smad ubiquitylation regulatory factor 1 (Smurf1). Creating a disturbance in the TGF-β1/BMP-7 pathways can effectively enhance the incursion and CSCs properties of malignant cells by upregulating the inhibitor of differentiation 1 (ID1) expression (Ning et al., 2021). Specifically, TAMs facilitate the development of CSC-like characteristics through TGF-β1-induced EMT, and they could potentially aid in the study of HCC prognosis (Fan et al., 2014). Targeting CSCs may, therefore, be a promising strategy for preventing HCC metastasis and improving patient outcomes.

### TAMs affect the proliferation of HCC cells

Long non-coding RNAs (lncRNAs) are a type of RNA that exceed 200 bases in length (Han et al., 2019). TAMs secrete exosomes containing M2 Mφ polarization associated lncRNA (lncMMPA). In HCC cells, lncMMPA has been found to interact with miR-548s, increasing the mRNA level of ALDH1A3, and induce cell glycolysis and proliferation (Xu et al., 2022). HBeAg, an antigen associated with HBV, resulted in an increased level of lncRNA MAPKAPK5_AS1 (MAAS) of M2-TAMs via boosting the N6-methyladenosine adjustment of MAAS through the action of methyltransferase-like 3. M2 macrophage-derived exosomes containing MAAS facilitated the stimulation of cyclin-dependent kinase 4 (CDK4), CDK6, and S-phase kinase-associated protein 2 transcription that were induced by the MYC proto-oncogene (c-Myc), via sustaining the c-Myc protein, leading to the facilitation of G1/S transition. The multiplication of HBV-positive HCC cells was attributed to this phenomenon (Tao et al., 2022). The levels of exosomes containing microRNA (miR)-375 derived from TAMs subjected to IL-2 (Exo^IL2−TAM^) were higher than those TAMs not subjected to IL-2 (Exo^TAM^). These exosomes could decrease HCC cells multiplication and metastasis and facilitate apoptosis both *in vivo* and *in vitro* (Chen et al., 2022).

Promoting M1-TAMs polarization increased propensity for cell killing and phagocytosis (Lujambio et al., 2013). Nonetheless, a single investigation revealed that M1-TAMs augmented the NF-κB p-p65/p65 ratio in HCC cells, thereby facilitating the nuclear translocation of p65. This, in turn, led to an elevated amount of malignant liver cells in the phases of the cell cycle known as S and G2/M, as well as the upregulation of CDK1, CDK2, and cyclin D1 expression. These studies indicated that M1-TAMs increased tumor cell proliferation. It is noteworthy that inhibiting the nuclear translocation of p65 brought on the reversal of alterations in the cell cycle, anti-apoptotic capacity, and protein expression induced by M1-TAMs of HCC (Sharen et al., 2022).

The M2-TAMs overexpressed IL-17 in HCC, particularly upon oxaliplatin treatment. Activation of the IL-17 receptor and lysosome-associated membrane protein 2A is crucial for chaperone-mediated autophagy induction by IL-17 in HCC cells. This, in turn, hinders apoptotic processes in response to oxaliplatin treatment (Guo et al., 2017). A significant increase in T cell immunoglobulin and mucin-domain containing protein-3 (Tim-3) expression was observed in TAMs of HCC patients. By activating NF-κB in Mφ, Tim-3 promotes IL-6 releasing, and the multiplication of liver cancer cells is, therefore, enhanced. However, receptor interacting protein 140 (RIP140), which is expressed in TAMs, exerts the opposite effect (Yan et al., 2015; Hu et al., 2017). STAT3 phosphorylation promoted cell proliferation and migration after IL-6 stimulation (Mano et al., 2013). Additionally, the progression of tumors in relation to alcohol consumption has been linked to a crucial mechanism involving IL6-STAT3 signaling (Zhao et al., 2019). The production of CXCL8 by TAMs resulted in an increase in the miR-17 cluster, comprising miR-18a and miR-19a. These two miRNAs were observed to stimulate tumor metastasis and cell proliferation in HCC and were related to elevated metastasis and prolonged survival in HCC patients during clinical investigations (Yin et al., 2017). According to a research study, the creation of insulin-like growth factor-1 (IGF-1) by M2-TAMs could boost the expansion and spread of HCC cells (Sprinzl et al., 2015).

Dectin-3, a C-type lectin receptor (CLR), is responsible for inducing the apoptosis of tumor cells and inhibiting their proliferation by regulating the glycolysis of Mφ. Mφ deficient in Dectin-3 were found to significantly facilitate the proliferation of H22 cells and suppress their apoptosis (Qu et al., 2022). Releasing complement C1q from Mφ present in the inflammatory milieu was regarded as an unorthodox mechanism for activating the β-catenin pathway in periportal hepatic progenitor cells, which results in the enlargement and de-differentiation (Ho et al., 2020). The finding highlights the function of Mφ in triggering the pathway activation in the hepatic progenitor cells. TAMs induce proliferation of HCC cells through these mechanisms. Targeting these pathways or inhibiting the secretion of TAMs cytokines and exosomes is an effective way to suppress the proliferation of HCC cells.

### TAMs have the potential to influence invasion, migration, and angiogenesis in HCC

EMT is responsible for inducing a temporary and reversible loss of differentiation in epithelial cells, leading to the development of a mesenchymal-like or mesenchymal phenotype (Giannelli et al., 2016). This allows them to detach from the original tumor and migrate to distant locations, leading to metastasis. Activated Mφ promotes the invasion, migration, and angiogenesis of HCC (Wu and Zheng, 2012). There are multiple signaling pathways that regulate EMT, like TGF-β and Wnt/β-catenin, and entail the activation of transcriptional regulators, namely, Snail, Slug, and Twist. Low expression of CD86^+^, overexpression of CD206^+^, and oncoprotein-induced transcript 3 (OIT3) in TAMs are significantly associated with tumor invasion abilities such as multifocal tumors and late-stage tumor lymph node metastasis (TNM) (Jiang et al., 2021; Yang et al., 2022). M2-TAMs can facilitate the process of EMT and proliferation in liver cancer (Li et al., 2018b; Xiao et al., 2018; Jiang et al., 2021), while M1-TAMs act opposite to the action of M2-TAMs (Zhang et al., 2020; Chen et al., 2021). Of note, CD68^+^ human leukocyte antigen (HLA)-DR^+^ M1-TAMs promoted carcinoma cell movement through the activation of NF-κB/focal adhesion kinase (FAK) signaling (Wang et al., 2014). During the polarization process of U937 Mφ from the M2-TAM to M1-TAM type, lnc-Ma301 was found to be overexpressed. This overexpression led to an interaction between lnc-Ma301 and caprin-1 and then ultimately inhibited the metastasis of HCC and EMT through the AKT/ERK1 signaling (Luo et al., 2021). M2-TAM leads to a significant increase in expressing the mesenchymal-associated markers N-cadherin and vimentin of malignant liver cells, along with Snail, Twist, and ZEB1. At the same time, it decreases the ratio of E-cadherin/N-cadherin in cancerous cells (Zhu et al., 2015; Li et al., 2019; Jiang et al., 2021). Carbonic anhydrase XII (CA12) expressed in TAMs stimulates the production of significant quantities of CCL8, which promoted cancer cell EMT (Ning et al., 2022). Additionally, M2-TAMs promote the process of EMT through paracrine releasing vascular endothelial growth factor (VEGF), IL-10, and CCL18 (Wang et al., 2017; Li et al., 2018c; Feng et al., 2018). The TGF-β1 secreted by TAMs initiated activate Gli2/IGF-II/ERK1/2 pathway in HCC cells (Liu et al., 2020). In addition, TGF-β1 inhibiting HCC miR-28-5p expression could be a vicious cycle (Zhou et al., 2016). The aforementioned signals were demonstrated to stimulate the multiplication, invasiveness, and migratory potential of HCC cells. M2-TAMs facilitated the attaching Smad2/3 to the miR-362-3p promoter, bringing about an upregulation of miR-362-3p, which was attributed to the release of TGF-β. The maintenance of EMT was regulated by miR-362-3p through the modulation of CD82, a significant member of the tetraspanin family (Zhang et al., 2019). M2-TAMs have been shown to release IL-1β, which stimulates the production of HIF-1α in cancerous cells through cyclooxygenase-2 (COX-2) (Zhang et al., 2018; Zhang et al., 2018; Gao et al., 2023). Additionally, IL-1 was shown to facilitate EMT in HCC cells via activating the IL-1R1/IκB/IKK/NF-κB pathway (Wang et al., 2020; Meng et al., 2022). Inflammatory cytokines secreted by activated macrophages can cause a reduction of E-cadherin expression in HCC. This is achieved through activating NF-κB/Slug signaling and destabilizing the E-cadherin/β-catenin complex. The instability of the E-cadherin/β-catenin complex was caused by phosphorylating β-catenin and E-cadherin with tyrosine kinases c-Src and EGFR (Lin et al., 2006; Wang et al., 2014). TAMs secreted CXCL12 to bind with CXCR4 of HCC cells, activating the ERK and AKT pathways, promoting HCC proliferation and metastasis (Song et al., 2021). Activated macrophages, known as TAMs, can trigger the EMT of HCC cells. This process is mediated by the JAK2/STAT3/Snail pathway, which is activated by the inflammatory cytokine IL-8 (Fu et al., 2015). The diminishment in the G protein-coupled receptor kinase 2 (GRK2) expression of M2-polarized macrophages, which are stimulated by β2-adrenoceptors (β2-ARs), induces the activation of the cyclic adenosine monophosphate (cAMP)/protein kinase A/cAMP response element binding protein and cAMP/IL-6/STAT3 signaling pathways. This decrease in GRK2 expression contributes to the release of associated cytokines, such as VEGF, MMP-9, and IL-6. Ultimately, this leads to the enhancement of malignant biological activity in cancer cells (Wu et al., 2019). MiR-15b expression was upregulated in M2-TAMs and transferred to HCC cells through EVs. This transfer of miR-15b in EVs suppressed the activation of the Hippo pathway through LATS1. LATS1-mediated ubiquitination and degradation of YAP1 were suppressed. The displacement of YAP1 to the nucleus stimulates the upregulation of oncogenes, leading to increased multiplication, invasion, and propagation of neoplastic liver cells (Li et al., 2021). The interplay between TAMs and EMT is intricate and can facilitate the advancement and metastasis of cancer. Targeting these processes can be a hopeful approach for cancer treatment.

In well-differentiated HCC, there exists a correspondence between the number of TAMs and tumor blood vessels (Fujita et al., 2014; Wang et al., 2020). TAMs are linked to the promotion of neovascularization, and the count of tumor micro-vessels (MVs) is related to carcinoma invasion and metastasis (Peng et al., 2005). One mechanism is the production of MMP-9 by TAMs, which contributes to tumor angiogenesis, extracellular matrix (ECM) remodeling, and cancer cell invasion through the ECM, ultimately promoting cancer cell metastasis (Wu et al., 2019; Wang et al., 2020). VEGF is generally regarded as one of the most significant angiogenic factors released by Mφ (Zhou et al., 2017). Abnormal angiogenesis was linked to the activation of platelets by tissue factor (TF) secreted by invading Mφ and endothelial cells. Tumoral angiogenesis is significantly influenced by HIF-1α, VEGF, and TF (Dupuy et al., 2003; Zang et al., 2018). In tumor tissues, the presence of M-CSF, also known as CSF1, promotes carcinoma growth and angiogenesis by a paracrine effect on the CSF1R (Awasthi et al., 2023). The absence of functional polarization, as indicated by the M0 signature and the heightened presence of CCL2 receptors such as CCR2 and CX3CR1, as well as pro-angiogenic factors, were observed. The M0 signature was found to be linked with adverse clinical results, while the expression of CCL2 was linked to YAP and the formation of vascular networks (Thomann et al., 2021). The promotion of TAMs on EMT and angiogenesis are all important processes in carcinoma growth and metastasis, and understanding the interactions between these processes may lead to the emergence of novel cancer treatments.

### TAMs possess the capability to impact hepatic fibrosis in HCC

Hepatic stellate cells are responsible for hepatic fibrosis by depositing extracellular matrix proteins. These cells are activated, in part, by TAMs. Exacerbation of liver fibrotic changes, as well as induction of neighboring epithelial cells transformed into HCC, occurs when the aging response in hepatic stellate cells, which relies on p53, is eliminated (Lujambio et al., 2013). Long-term alcohol abuse facilitates the TAMs infiltration in HCC, exacerbating inflammation, fibrosis, and EMT in the disease process of HCC (Yan et al., 2017). *In vitro* experiments have demonstrated that glucagon-like peptide-1 (GLP-1) can suppress the proinflammatory characteristics of Mφ (Kawakubo et al., 2020). M2-TAMs engage in the procedure of hepatic fibrosis in HCC advancement and are managed via the PI3K-AKT-mTOR signaling (Zhang et al., 2022). Activation of the Notch signaling pathway in Mφ promotes hepatic fibrosis by upregulating NF-κB via cylindromatosis (CYLD) (He et al., 2015). In TAMs with autophagy defects, mitochondrial ROS enhances the NF-κB signaling and increases the releasement of IL-1α/β. NOX enzymes were the principal source of ROS. NOX1 and secretion of IL-1α/β and TNF-α facilitated the advancement of hepatic fibrosis and inflammation (Amano et al., 2017; Sun et al., 2017; Vandierendonck et al., 2021). Targeting the phenotype of TAMs and the cytokines they secrete is the key to blocking the progression of the fibrotic liver into HCC (Table 1).

**TABLE 1:** Mechanisms of TAMs influencing the progression of HCC. (↑ means promoting the process to promote HCC progression, and ↓ means inhibiting HCC progression by inhibiting the process.)

Effect on HCC progression	Mechanisms in TAMs	Mechanisms in carcinoma cells
Cancerous cell stemness↑	Secretion of CXCL1 and CXCL2	Upregulation of the expression of BCL-2 and downregulation of BAX and BAD
Cancerous cell stemness and proliferation↑	Expression of 5-LOX, production of LTB and LTC/D/E	Activation of ERK1/2
Cancerous cell stemness, proliferation, migration, and invasion↑	Secretion of IL-6	Activation of STAT3
Cancerous cell stemness↑	Secretion of S100A9	Activation of NF-κB
Cancerous cell stemness, migration, and invasion↑	Secretion of TNF-α	Activation of Wnt/β-catenin
Cancerous cell stemness, migration, and invasion↑	Secretion of CCL17	Activation of TGF-β1 and Wnt/β-catenin
Cancerous cell stemness, migration, and invasion↑	Secretion of exosome contained miR-17-92	Targeting Smurf1to break the balance of TGF-β1/BMP-7
Cancerous cell proliferation↑	Secretion of exosome contained MAAS	Stability of c-MYC, transcriptional activation of CDK4, CDK6, and S-phase kinase associated protein 2, and facilitation of G1/S transition
Cancerous cell proliferation ↑or↓	-	Upregulation of the NF-κB p-p65/p65 ratio and the expression of CDK1, CDK2, cyclin D1, or inhibition of nuclear translocation of P65
Cancerous cell proliferation↑	Secretion of IL-17	Activation of IL-17R and lysosome-associated membrane protein 2A
Cancerous cell proliferation, migration, and invasion↑	CXCL8	Upregulation of the miR-17 cluster (miR-18a and miR-19a)
Cancerous cell proliferation↑	IGF-1	-
Cancerous cell proliferation↑	Dectin-3 regulating the glycolysis of macrophages	-
Cancerous cell proliferation↑	Secretion of complement C1q	Activation of Wnt/β-catenin
Cancerous cell motility↑	-	Activation of NF-κB/FAK
Cancerous cell invasiveness and migratory potential↓	Overexpression of lnc-Ma301	Inhibition of AKT/ERK1
Cancerous cell invasiveness and migratory potential↑	High expression of CA12 and secretion of CCL8	Activation of EMT
Cancerous cell invasiveness and migratory potential↑	Secretion of VEGF, IL-10, and CCL18	Activation of EMT
Cancerous cell invasiveness and migratory potential↑	Upregulation of the expression of TGF-β1	Activation of the Gli2/IGF-II/ERK1/2 pathway
Cancerous cell proliferation, invasiveness, and migratory potential↑	Secretion of TGF-β1	Inhibition the miR-28-5p expression
Cancerous cell invasiveness and migratory potential↑	Secretion of TGF-β	High expression of miR‐362‐3p maintained EMT
Cancerous cell invasiveness and migratory potential↑	Secretion of IL-1β	Upregulation of HIF-1α facilitated EMT
Cancerous cell invasiveness and migratory potential↑	Secretion of IL-1	Stimulation of IL-1R1/IκB/IKK/NF-κB facilitated EMT
Cancerous cell invasiveness and migratory potential↑	Secretion of inflammatory cytokines	Activation of NF-κB/Slug and destabilization of the E-cadherin/β-catenin complex
Cancerous cell proliferation and migratory potential↑	Secretion of CXCL12	Activation of ERK and AKT
Cancerous cell proliferation and tumor angiogenesis↑	Secretion of CSF1	Activation of CSF1/CSF-1R
Cancerous cell proliferation, invasiveness, and migratory potential↑	Secretion of EVs contained miR-15b	Inhibition of the Hippo pathway by targeting LATS1
Tumor angiogenesis↑	Secretion of MMP-9	Remodeling ECM
Tumor angiogenesis↑	Secretion of CCL2	YAP protein
Hepatic fibrosis↑	Downregulation of the expression of CYLD	Activation of NF-κB
Hepatic fibrosis↑	Secretion of IL-1α/β and TNF-α	-

### Interplay between TAMs and other immune cells reshapes the immune microenvironment in HCC

The TME contains various immune cells, such as TAMs, T cells, B cells, and NK cells, whose secretion of cytokines leads to cellular interactions and ultimately reshapes the immune microenvironment, affecting the progression of the tumor. The TME often experiences hypoxia, a common phenomenon in various cancer types. This hypoxia can negatively impact cytotoxic T cells’ actions and promote recruiting regulatory T cells (Tregs), ultimately inducing a reduction in the tumor’s immunogenicity (Wu et al., 2022). In hypoxic tumor microenvironments, Tregs can impair the function of cytotoxic T cells. This is due to the induction of TREM1 in TAMs by HIF-1α under hypoxic conditions. The increase in TREM1 activates the ERK/NF-κB pathway as a reaction to oxygen starvation and cancer-specific metabolites, resulting in the increased expression of CCL20. This leads to the accumulation of CCR6^+^Foxp3^+^ Tregs (Wu et al., 2019). CD69^+^ T cells could trigger TAMs to produce indoleamine 2,3-dioxygenase (IDO), while TAMs can secrete IL-12 to activate T cells. Furthermore, IL-12 can also enhance the expression of IDO, which subsequently brings about Tregs expansion and the suppression of T cells’ reactions and proliferation (Zhao et al., 2012; Ye et al., 2016). The presence of CD74^+^ Mφ in HCC was discovered to have a correlation with more CD8^+^ cytotoxic T lymphocyte (CTL) infiltration, which displayed improved effector capabilities (Xiao et al., 2022). CD169^+^ Mφ were observed to significantly augment the multiplication, cytotoxic potential, and cell secretory product generation of CD8^+^ T cells in a manner relying on CD169. The downregulation of CD169 expression in these cells is linked to autocrine TGF-β secretion. This is opposite to the survival prognosis tendency of the entire amount of CD68^+^ macrophages (Zhang et al., 2016). In non-responders, the TME is typical to the close proximity of CD8^+^ T cells and Arg1^hi^ TAMs, rather than CD4^+^ T cells (Mi et al., 2022). M2-TAMs suppress the activity of NK cells (Xu et al., 2021).

**FIGURE 3:**
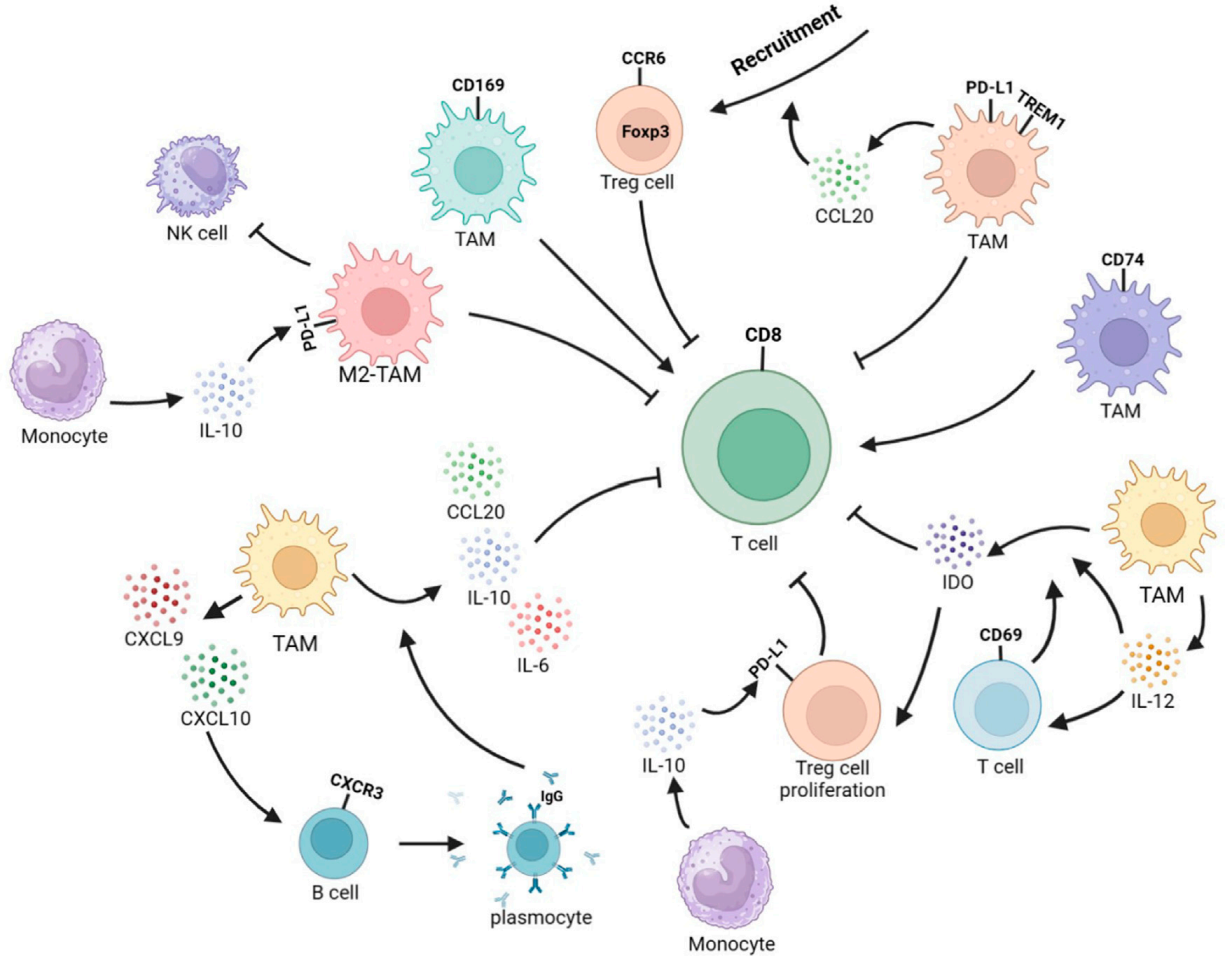
Interplay between TAMs and other immune cells reshapes the immune microenvironment in HCC. TREM1^+^TAMs secrete CCL20 to recruit CCR6^+^Foxp3^+^ Tregs and exert immunosuppressive function. CD69^+^ T cells can stimulate TAMs to produce IDO, and TAMs can secrete IL-12 to activate T cells. In addition, IL-12 can enhance IDO expression, leading to Treg expansion and inhibition of T-cell response and proliferation. CD74^+^ TAMs induce stronger effector function in CD8^+^ CTLs. CD169^+^ TAMs significantly enhance the proliferation, cytotoxic potential, and secretion of CD8^+^ T cells in a CD169-dependent manner. M2-TAMs can to inhibit NK-cell activity. Monocytes-secreted IL-10 promotes PD-L1 expression in Tregs and TAMs. TAMs could secret CXCL9 and CXCL10. CXCL10 stimulates CXCR3^+^B cells to differentiate into IgG-producing plasma cells. IgG stimulates Fc receptors on TAMs, leading to the secretion of IL-10, IL-6, and CCL20 to inhibit the recruitment and function of CD8^+^ T cells.

As a type of co-inhibitory molecule, PD-L1 can bind to the programmed death-1 (PD-1) receptor on the surface of T cells to regulate immune responses and inhibit immune cells from killing tumor cells, and is closely related to T-cell exhaustion and tumor immune evasion. PD-L1 can be expressed in tumor cells, TAMs, and vascular endothelial cells, but PD-L1 expression was predominantly witnessed on CD68^+^ TAMs rather than HCC cells in the TME. Additionally, high expression of PD-L1 was detected on TREM-1^+^ TAMs (Wu et al., 2019; Park et al., 2021). Abnormal levels of cytokines such as HGF, VEGF, TNF-α, and IL-6 are often detected in the serum of HCC patients and are closely related to liver cancer prognosis. Among them, elevated levels of IL-6 in serum lead to the downregulation of protein tyrosine phosphatase receptor O (PTPRO) expression in monocytes and Mφ. A depletion of PTPRO mediates high expression of PD-L1 in monocytes and Mφ through the JAK2/STAT1 and JAK2/STAT3/C-MYC pathways (Zhang et al., 2020). Changes in the expression of enzymes related to abnormal glycolipid metabolism can also affect the expression of PD-L1 in TAMs. The overexpression of key glycolytic enzyme PFKFB3 in the monocytes of the TME induces high expression of PD-L1 through mediation of the NF-κB signaling pathway (Chen et al., 2019). Monocytes upregulate IL-10 secretion through the expression of lipid-binding protein FABP5 and inhibition of the PPARα pathway, and IL-10 can promote the expression of PD-L1 in Tregs and TAMs (Liu et al., 2022), ultimately resulting in immunosuppression. Abnormal metabolism of tumor cells can lead to increased secretion of IgA. Stimulation of IgA signaling induces M2 polarization and upregulates PD-L1 expression through YAP, leading to reduced CD8^+^ T-cell infiltration (Sung et al., 2022). Upregulating PD-L1 in TAMs was also found to be mediated by STAT3, which is induced by tumor-derived Sonic hedgehog (Shh) signaling (Petty et al., 2021). Tumor cells activate the hedgehog (Hh) signaling pathway to suppress Mφ to produce CXCL10 and CXCL9 (Petty et al., 2019). CXCL10 secreted by Mφ attaches to CXC motif chemokine receptor (CXCR)3 on B cells, causing them to differentiate into IgG-producing plasma cells. IgG stimulates Fc receptors on Mφ, which in turn release cell secretory product like IL-10, IL-6, and CCL20, causing a diminish in the recruitment and response of antineoplastic CD8^+^ effector cells (Wei et al., 2019). The secretion of CXCL9 by TREM2^+^ TAMs was reduced, while Galectin-1 (Gal-1) secretion was increased. This resulted in PD-L1 highly expressing in vascular endothelial cells, which impeded the recruiting and infiltration of CD8^+^ T cells into the TME (Tan et al., 2023). The high levels of DNA damage in HCC cells activate the cyclic GMP–AMP synthase (cGAS)–stimulator of interferon genes (STING) signaling, leading to PD-L1 expression in M1-TAMs through the STING-IRF3-STAT1 pathway. This promotes immunosuppression, facilitating oncogenesis and malignant development. Additionally, M1-TAMs with a stimulated cGAS–STING signaling can enlist T lymphocytes via the STING-IRF3 signaling (Ma et al., 2023). The IL-27 secreted by Mφ leads to the phosphorylation of STAT1 and reproduces an IFN-γ-like reaction in cancer cells, which is similar to the effect of IFN-γ, leading to the upregulation of TAP2 and MHC-I proteins, thereby increasing the immune clearance rate. Simultaneously, it induces T-cell exhaustion by promoting PD-L1 expression and IDO secretion (Rolvering et al., 2018). Furthermore, as antigen-presenting cells, the infiltration of MHC II^low^ TAMs is positively correlated with the progression of HCC (Wang et al., 2011). The overexpression of PD-L1 in HCC is also related to the inhibition of PD-L1 ubiquitination and degradation processes in tumor cells and TAMs. Golgi membrane protein 1 (GOLM1) in HCC cells inhibits the ubiquitination and degradation of PD-L1 by COP9 signalosome 5 and promotes the secretion of EVs containing PD-L1 in HCC cells, upregulating the expression of PD-L1 in TAMs (Chen et al., 2021). The lack of GSK3β in TAMs can also inhibit the ubiquitination and degradation of PD-L1 (Sun et al., 2022). On one hand, high expression of PD-L1 can reshape the immune inhibitory microenvironment. However, on the other hand, increased levels of PD-L1 expression can facilitate the treatment of PD-1 inhibitors (Figure 3).

## Conclusion

TAMs originate from peripheral blood monocytes, M-MDSCs, and KCs. The cytokines and EVs secreted by tumor cells, stromal cells including TAMs, and other interstitial cells within the tumor tissue can induce the recruitment of peripheral monocytes. Meanwhile, TAMs in the tumor tissue can also increase their infiltration by proliferation. Altered glucose and lipid metabolism in HCC and cytokines secreted by tumor cells induce Mφ polarization and functional phenotype changes. However, due to the influence of the TME, TAMs often exert immunosuppressive functions, inducing the occurrence of malignant behavior in HCC. TAMs directly activate or inhibit the NF-κB, IL-6/STAT3, Wnt/β-catenin, TGF-β1/BMP, and ERK1/2 signaling pathways in HCC cells by producing cytokines and exosomes and overexpressing related proteins, affecting carcinoma cell proliferation, invasion and migration ability, angiogenesis, and liver fibrosis progression, thus affecting the progression of HCC. Additionally, the coordination between TAMs and immune cells reshapes the immune-suppressive microenvironment within cancer. Among them, elevated PD-L1 expression in TAMs suppresses the stimulation of CD8^+^ T cells, induces CTL exhaustion, and promotes Treg recruitment, which is one of the key mechanisms for inhibiting CD8^+^ T-cell killing of HCC cells. The resistance mechanism of the small-molecule targeted drug sorafenib used in HCC treatment is also linked with TAMs. Therefore, focusing on the mechanism of TAMs interacting with tumor and immune cells in HCC helps to target relevant pathways to inhibit the progression of HCC.
